# Progress of preoperative and postoperative radiotherapy in gastric cancer

**DOI:** 10.1186/s12957-018-1490-7

**Published:** 2018-09-13

**Authors:** Nan Zhang, Qian Fei, Jiajia Gu, Li Yin, Xia He

**Affiliations:** 1 Department of Radiation Oncology, Jiangsu Cancer Hospital & Jiangsu Institute of Cancer Research & Affiliated Cancer Hospital of Nanjing Medical University, Nanjing Medical University Affiliated Cancer Hospital, 42 Baiziting, Nanjing, 210009 Jiangsu China; 2 The Fourth Clinical School of Nanjing Medical University, Nanjing, China

**Keywords:** Gastric carcinoma, Radiotherapy, Radiation field, D2 lymph node dissection

## Abstract

**Background:**

Gastric carcinoma, a highly common malignant tumor, is treated mainly by surgery. Meanwhile, radiotherapy is attracting increased attention as a crucial locoregional therapy. However, the application of radiotherapy in gastric carcinoma is still limited and radiation standards remain debatable.

**Main body:**

The use of preoperative radiotherapy for treating gastroesophageal junction cancer has advanced. However, additional phase III clinical trials are needed to further verify the therapeutic value of preoperative radiotherapy for gastric cancer. Patients with D1 or D1 plus lymphadenectomy can benefit from postoperative radiotherapy obviously, and postoperative radiotherapy may be effective for patients with D2 lymphadenectomy with a high N stage. The target volume delineation of preoperative and postoperative radiotherapy should be based on clinical experience and the characteristics of lymphatic drainage.

**Conclusions:**

With the advancement of radiotherapy technology, preoperative and postoperative radiotherapy are becoming increasingly accepted as important auxiliary treatments for gastric cancer.

## Background

The morbidity of gastric cancer has been declining worldwide but remains a highly common malignancy, which is the third leading cause of cancer-related mortality. In 2012, one million new cases occurred around the world, with more than 72,000 deaths [1]. More than 75% newly diagnosed patients were in an advanced stage because of the lack of the typical clinical premonitory symptoms of gastric cancer. Advanced stage means the tumor has invaded the muscle layer or lymph node, and the survival rates of patients in this stage are only 20–50% [2]. Among patients with advanced stage gastric cancer, approximately 50% have lost the chance of surgery. Therefore, comprehensive treatment based on radiotherapy (RT) and chemotherapy has recently received much attention.

In recent years, the application of RT in gastric cancer has become increasingly common with the development of radiation technology. In 2001, John et al. published the results of the INT0116 trial in the New England Journal and caused the RT change from the traditional palliative treatment to important adjuvant therapy in the multidisciplinary treatment for gastric cancer [3].

## Preoperative RT

Preoperative RT is mainly used to reduce tumor burden in patients with advanced gastric cancer. This process enables inoperable patients to be eligible for operation. In addition, preoperative RT may play a unique role in controlling micrometastasis, and the pathological response after preoperative RT may provide important prognostic information [4, 5]. The results of several major clinical trials showed that gastroesophageal junction (GEJ) cancer achieves a better therapeutic effect than that of gastric cancer in terms of preoperative RT (Table 1).

**Table 1:** Preoperative RT III clinical trials

Study/institute	*n*	Tumor location	Groups	Local control	Survival
1998 Zhang et al. [6]	370	EGJ	RT+S vs. S	Local control and local recurrence rate 61.4% vs. 51.7%	10-year OS 20.3% vs. 13.3%
2009 Stahl et al. [7]	119	EGJ	CRT+S vs. C+S	Pathological complete response rate 15.6% vs. 2.0%	3-year OS 47.4% vs. 27.7%
2012 Van Hagen et al. [8]	366	EGJ or EC	CRT+S vs. S	Local recurrence rate 14% and 34%	5-year OS 47% vs. 34%
2002 Skoropad et al. [9]	102	GC	RT+S vs. S	No sense	No sense

*EGJ* esophagogastric junction, *GC* gastric cancer, *EC* esophagus cancer, *RT* radiotherapy, *CRT* concurrent radiotherapy, *S* surgery

In 1998, Chinese researchers found that 370 patients with GEJ cancer treated with preoperative RT significantly improved in tumor resection rate relative to the patients treated with surgery alone (89.5% vs. 74.9%). The local control rates in the two groups were 61% and 45% (*P* < 0.05), and the 10-year survival rates were 20.3% and 13.3% (*P* = 0.009), respectively. These results indicate that preoperative RT may be beneficial for improving the local control rate and the overall survival (OS) of patients with GEJ cancer [6]. Stahl revealed that preoperative RT significantly improved the rate of pathological complete response (15.6% vs. 2.0%) of GEJ adenocarcinoma and increased the 3-year OS rate (47.4% vs. 27.7%, *P* = 0.07) [7]. Similarly, Hagen and co-workers investigated 366 cases of gastric cancer or GEJ cancer and found that the patients treated with preoperative radiochemotherapy (carboplatin + paclitaxel, 5 weeks; 41.4 Gy/23 f, 5 days/week) attained a significantly improved rate of tumor resection (92% vs. 69%, *P* < 0.001) and OS (49.4 months vs. 24 months, median survival) relative to those of the patients treated with surgery alone. Besides, preoperative radiochemotherapy was related to a decreased rate of local recurrence (LRRs, 14% and 34%, *P* < 0.001) and distant metastases rates (29% and 35%, *P* = 0.025) relative to surgery [8]. In this study, the regimen above became the recommended treatment program for GEJ adenocarcinoma in the USA.

Compared with the progress of preoperative RT in treating GEJ cancer, the application of preoperative RT still lacks large-scale phase III clinical trials for gastric cancer. In 2002, Skoropad investigated 102 cases of resectable gastric cancer and found that preoperative RT (20 Gy/5 f) did not significantly improve the local control and long-term survival relative to surgery alone (20-year OS rates, 32% and 18%, *P* = 0.555) [9]. In this study, the irradiation technology was regressive despite the follow-up time of 20 years, and the number of cases was minimal. Conversely, some single-arm prospective trials and retrospective analyses confirmed that adopting preoperative chemoradiotherapy (CRT) appeared safe and beneficial for advanced gastric cancer patients [10–16]. Kumagai presented results indicating that patients with gastric or GEJ cancer treated with preoperative RT or CRT attained a higher rate of survival than those patients treated with surgery alone. Simultaneously, he found that adding preoperative RT or CRT did not significantly decrease the rates of postoperative recurrence and mortality [15].

The response of preoperative chemotherapy in gastric cancer has been universally accepted, but whether preoperative concomitant radiochemotherapy can offer survival benefits is unclear relative to preoperative chemotherapy alone [17–19]. The ongoing TOPGEAR clinical trial is designed to address this issue [20]. In addition, a phase II clinical trial (NCT02301481) is being conducted by the Chinese Academy of Medical Sciences to determine whether preoperative radiochemotherapy is superior to preoperative chemotherapy alone for advanced gastric adenocarcinoma patients. A similar clinical study (NCT01815853) is conducted at the Sun Yat-sen University to observe the OS and safety of the preoperative radiochemotherapy. These results of the clinical trials are promising [21].

## Postoperative RT

The findings of the INT0116 trial show the important role of postoperative RT in the adjuvant treatment of gastric cancer [3]. However, many deficiencies, such as the lack of the strict control of surgical type (only 10% cases of D2 dissection), the backwardness of radiation technology, and the low treatment compliance, are observed in research. After the Lancet published the 15-year follow-up results of D1 and D2 dissection in 2010, the advantage of reducing the local recurrence caused D2 to gradually become the standard surgical operation of resectable advanced gastric cancer [22]. The following section is mainly based on the current situation of postoperative RT after D2 dissection. The main results of phase III clinical trials in recent years are displayed in Table 2.

**Table 2:** Postoperative RT III clinical trials

Study/institute	*n*	D2	RT	pN+	III-IV	DFS/RFS	OS	Remarks
2001 INT0116 USA [3]	556	10%	2D	85%	NR	3-year 48% vs. 31% (*p* < 0.001)	3-year 50% vs. 41% (*p* = 0.005)	
2012 INT0116 [23]						10-year similar	10-year similar	D1 and D2 benefit
2012 ARTIST South Korea [24]	458	100%	2D or 3D	86%	41%	3-year 78% vs. 74% (*p* = 0.0862)	NR	N^+^DFS benefit
2015 ARTIST Final report [25]						5-year 74% vs. 68% (*p* = 0.092)	5-year 75% vs. 73% (*p* = 0.527)	N^+^ and GC DFS benefit
2012 NCC South Korea [26]	90	100%	2D or 3D	98%	100%	5-year 65% vs. 55% (*p* > 0.05)	5-year 65% vs. 55% (*p* > 0.05)	LRRFS and III stage DFS benefit
2012 IMRT China [27]	351	100%	NR	86%	71%	5-year 45% vs. 36% (*p* = 0.029)	5-year 48% vs. 42% (*p* = 0.122)	

*NR* not reported, *OS* overall survival, *DFS/RFS* disease-/relapse-free survival, *LRRFS* locoregional failure-free survival, *GC* gastric cancer

In 2012, the 10-year follow-up results of INT0116 showed that postoperative concurrent radiochemotherapy continued its survival benefit and that either the D1 or the D2 subgroup can benefit from this modality [23]. Simultaneously, researchers from South Korea presented results of the ARTIST trial, which showed that the postoperative RT did not significantly improve the rate of disease-free survival (DFS), but for patients with pathologic positive lymph nodes, the postoperative RT demonstrated its survival benefits with no statistical significance (*P* = 0.38) [24]. The final results of the ARTIST trial after a 7-year follow-up also yielded similar conclusions [25]. In 2012, two other phase III clinical trials from Korea and China revealed that the postoperative RT after D2 dissection did not improve OS but enhanced the rate of local recurrence-free survival [26, 27]. In 2017, Stumpf et al. analyzed 3656 patients with resected gastric adenocarcinoma from the National Cancer Database in 2004 to 2012 and compared the OS rates between the perioperative chemotherapy group and the postoperative adjuvant radiochemotherapy group. The results of univariate and multivariate analyses suggested that the OS rates in the postoperative adjuvant radiochemotherapy group were superior to those of the perioperative chemotherapy group. In the subgroup analysis, the patients with positive surgical margins benefited more with adjuvant RT [28].

Given the analysis of the above phase III clinical trials, the trend toward negative results for the three trials, which are from the east, may be explained by the wide use of D2 dissection and postoperative chemotherapy. The lymph node dissection is substantial in D2. Hence, for some patients, treatment with D2 dissection plus postoperative chemotherapy is sufficient. On the contrary, the positive results of INT0016 were mainly for the vast majority of patients treated with D1 dissection, in which the lymphatic dissection range is minimal; thus, postoperative RT can play an important role in terms of local control. Hence, patients with gastric cancer require being screened before receiving postoperative RT [1].

In our opinion, whether patients require adding RT after D2 dissection should be determined by the disease stage. Sasako found that for patients with high-stage gastric cancer, postoperative chemotherapy alone cannot improve the RFS [29]. Therefore, adding RT for stage III gastric cancer patients after D2 dissection is necessary. In the ARTIST trial, only 41% of the patients were diagnosed at the III–IV stage, and postoperative RT may be an over-treatment for patients with stages I–II, where postoperative chemotherapy alone was sufficient. Therefore, the patients with pathologically positive lymph nodes in the ARTIST trial did not significantly improve in DFS, whereas for the patients with higher stages, especially stage III, the advantages of postoperative RT for local control were prominent. In an American retrospective review of 23,461 patients with early gastric cancer (IB–II) treated with postoperative RT, Datta concluded that patients in all stages of early gastric cancer can acquire survival benefits [30]. However, the researchers did not clarify whether the surgical patients were treated with D1 or D2 dissection. Well-designed prospective randomized clinical trials are still required to validate whether patients in different stages of gastric cancer with pathologic positive lymph nodes can benefit from postoperative RT.

The latest gastric cancer NCCN guidelines (2018.V1) still recommend postoperative chemotherapy after D2 lymphadenectomy, and postoperative CRT is preferred for surgical patients with a range of resection less than D2. The ARTIST II trial is a phase III randomized trial of adjuvant chemotherapy with compound tegafur–oteracil potassium capsules (S-1) versus S-1/oxaliplatin ± RT for surgical patients with positive nodes [31]. Remarkably, the results of the phase II clinical trial based on S-1 and cisplatin showed that the postoperative concurrent CRT group improved 3 years of DFS relative to the postoperative chemotherapy group, and the toxicities were acceptable [32]. In recent years, several meta-analyses have demonstrated the role of perioperative RT in treating gastric cancer [33–35].

In summary, RT can be used as an important adjuvant therapy during the perioperative period of patients with surgical gastric cancer in an advanced stage, especially for some specific patients after D2 dissection, which effectively improves the PFS and reduces the rate of local recurrence. The value of preoperative RT in gastric cancer still requires further validation, and we anticipate further results of relevant randomized controlled clinical trials. In addition, the screening of tumor-derived radiosensitivity markers has attracted increasing attention in recent years. For example, positive E2F-1 expression and negative HER2 expression may indicate that the patients with gastric cancer treated with postoperative CRT will achieve a good outcome, and in vitro studies have shown that CHK1 overexpression may be associated with radiation resistance [36–38]. Therefore, these markers can be assumed to be used as new risk factors for predicting the survival outcome of gastric cancer patients to select those who may benefit from the perioperative period RT.

## Progress of treatment volume range

### Preoperative target volume

Previous preoperative target volume includes the whole stomach and large node areas (paraesophageal, extending from the trachea for bifurcation and the lesser curvature of the stomach to the posterior second thoracic vertebra) because of a lack of consolidated phase III clinical trials to define the target volume of gastric cancer. In 2009, EORTC-ROG (European Organization for Research on the Treatment of Cancer) redefined the CTV of GEJ adenocarcinoma and gastric adenocarcinoma, which reduced the error [39]. The therapeutic efficacy of the preoperative RT in gastric cancer has not reached a consensus; therefore, we only analyzed the target volume of GEJ cancer or proximal gastric cancer in this study.

The stomach is a hollow organ, and its position may be influenced by respiratory motion and body movement. The reduction of error of CTV caused by swinging and breathing has become our primary task. Stahl assumed that the CTV includes a 5-cm margin of the proximal primary tumor, a 3-cm margin of the distal primary tumor, and a 1-cm margin of all nodal areas at risk [7]. The PTV margin of 8 mm expanded in all directions from the CTV to reduce the systematic error and target displacements. Hagen et al. defined PTV as the 4-cm margin of the primary tumor [8]. In 2009, the expert opinions of specialists in EORTC-ROG highlighted their definition of PTV as the 1-cm margin of the proximal and transverse CTV, which is the 1.5-cm margin of the distal CTV, to reduce the error; this definition is similar to those of the above two studies, given the lack of sufficient evidence to set the criteria for the target volume [39].

In accordance with Siewert’s classification, the opinions of specialists in EORTC-ROG (2009) proposed a lymphatic drainage in different types of GEJ cancer; this proposal provided clinicians with a reference for delineating the target volume. However, the opinions failed to combine with computed tomography (CT) scans or other radiographic studies. Additionally, the patterns of local regional recurrence for gastric cancer were disregarded.

In 2014, on the basis of the study of Hagen, Oppedijk suggested patterns of recurrence for esophageal or GEJ cancer after preoperative radiochemotherapy [40]. After at least 24 months of follow-up, the overall recurrence rates of the surgery group and the CRT plus surgery group were 58% and 35%, respectively. The LRR of the CRT+S group reduced from 34 to 14%. A total of 5% of the patients of the preoperative CRT group experienced local relapse within the irradiated field; 2% experienced a local relapse at the edge of the irradiated field; 6% experienced local relapse outside the irradiated field. In this study, disease relapse mainly occurred in the celiac lymph nodes, para-aortic lymph nodes, and peritoneum, which were associated with the distal esophagus and esophagogastric junction (EGJ) cancer. However, the patients with EGJ cancer only constituted one-fourth, which may have an implication in delineating the high-risk areas of recurrence in EGJ cancer.

Oppedijk found that the incidence of local relapse outside the irradiated field remains high, and expanding the target volume for preoperative RT is necessary. Furthermore, we still lack reliable evidence for delineating the clinical target volume for preoperative RT in GEJ adenocarcinoma, and large-scale clinical trials on regional lymph node recurrence and failure modes are required.

### Postoperative target volume

Developing a uniform standard for the delineation of the postoperative RT target volume is difficult because of the different sites, stages, and lymphatic metastases in gastric cancer; the various surgical methods, and the dissimilar conditions of postoperative cutting edge. The earliest guideline for defining the target volume for postoperative RT that was based on the primary tumor sites and the pathway of lymph node metastasis was proposed by Smalley and Tepperin [41, 42]. However, the guideline was recommended for D1 or D1 + lymphadenectomy and during the era of 2D RT techniques with adverse reactions and low local control rates. This article mainly discusses the target volume after D2 lymphadenectomy.

Nam retrospectively analyzed 291 patients after D2 dissection. A total of 83 target volumes of patients included the gastric stump, whereas the remaining 208 did not. The results showed that no significant differences in 5-year OS and DFS existed between these two groups. However, 3–4 grade diarrhea was more common in the patients with target volumes that included the gastric remnant. Therefore, Nam suggested that the target volume should exclude the gastric stump for patients treated with D2 dissection [43]. As found previously, the ARTIST trial also excluded the gastric stump from the irradiated field. Aside for the temporary adverse effects, the long-term survival of patients, especially the occurrence of gastric stump cancer, must also be monitored. Ohira found that the average interval of occurrence of gastric stump cancer was 6.8–18.8 years, but the follow-up period was only 5 years in the study of Nam [44]. The occurrence of postoperative gastric stump carcinoma should be of particular concern, although no report has explored the relationship between postoperative RT and gastric stump cancer. Furthermore, the study of Nam adopted the traditional 2D RT with added adverse reactions, but modern radiation technology has a unique advantage in reducing adverse reactions. In the NCC trial, the patients with irradiated fields that included the gastric stump obtained a high dose in the left renal area. Another study from China, namely, the intensity-modulated radiation therapy (IMRT) trial, demonstrated that with advanced IMRT technology, the toxic side effects caused by radiation exposure to the remnant stomach can be controlled.

**Fig. 1:**
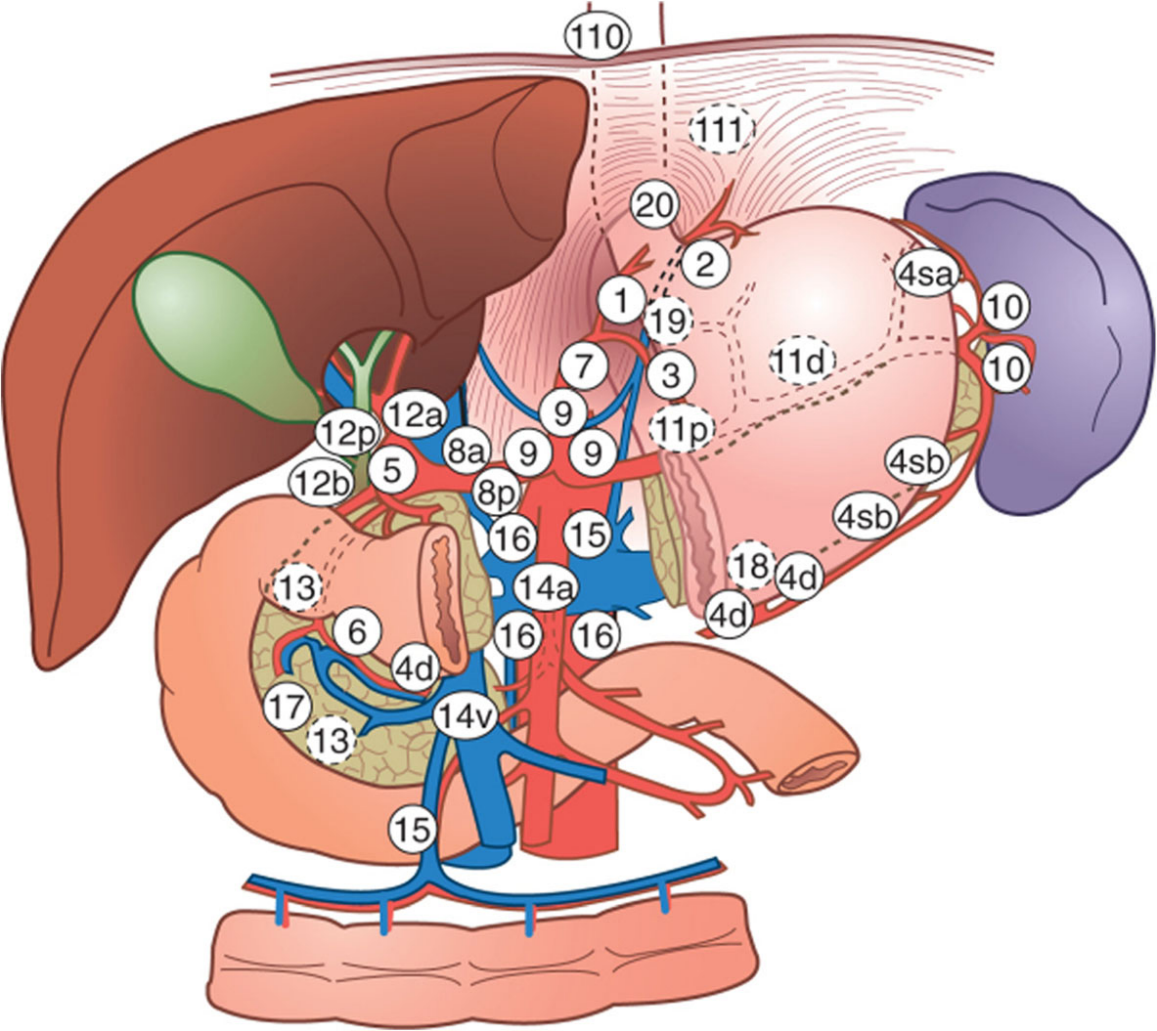
Schematic diagram of lymph node station. LN, lymph node; 1 right cardiac nodes; 2 left cardiac nodes; 3 nodes along the lesser curvature; 4 nodes along the greater curvature; 5 suprapyloric nodes; 6 infrapyloric nodes; 7 nodes along root left gastric artery; 8 nodes along common hepatic artery; 9 nodes around celiac axis; 10 nodes at splenic hilum; 11 lymph nodes along the proximal SA; 12 nodes at the hepatoduodenal ligament; 13 nodes on the posterior surface of the pancreatic head; 14 lymph nodes along the SMA or superior mesenteric vein; 15 nodes along the middle colic vein; 16a lymph nodes around the abdominal aorta for the upper margin of the celiac trunk to the lower margin of the LRV; 16b lymph nodes around the abdominal aorta from the upper margin of the LRV to the aortic bifurcation; 110 lymph nodes in the lower thoracic paraesophageal; 20 lymph nodes in the esophageal hiatus of the diaphragm [46]

Except for the ARTIST trial, almost all the phase III trials defined node nos. 1–16 as node areas at risk (Table 3, Fig. 1). The node areas at risk in the ARTIST trial only included node nos. 7–9 and 12–16, which received a reduced dose exposure to the intestinal tract. Besides, no difference existed between surgery plus RT and chemotherapy alone in adverse reactions. This result implies that the traditional node areas at risk may be exceedingly large for gastric cancer. Selective RT to high-risk lymph nodes should agree with the patterns of lymph nodes (LNs) recurrence after D2 dissection, which further optimizes the target volume. In 2012, Chang retrospectively investigated 357 gastric cancer patients with stage III after D2 or D3 dissection [45]. The results showed that the peritoneum was the most common site of recurrence, and the most common recurrent LNs was outside the field of D2 dissection (node nos. 12–16). Node nos. 16a as well as 16b are the most common recurrent lymph node whatever the site of primary tumor is.

**Table 3:** Postoperative RT III clinical trials, toxic reactions, and target volume

Study/institute	RT dose (Gy)	Intervention	Severe toxicity	Target volume	Completed rate
2001 INT0116 USA [3]	45	CRT, 45Gy, 5FU+LV	Grade 3+, 41%, Grade 4+,32%	Tumor bed, regional node (nos. 1–16)	63%
2012 ARTIST South Korea [24]	45	CT-CRT-CT, CRT: Capecitabine; CT: XP	Similar to chemotherapy alone	Tumor bed in T4 LN (nos. 7–9 and 12–16)	82%
2012 NCC South Korea [26]	45	CRT, 5FU+LV	Grade 3+ hematologic toxicities; 20% vs. 25% G3+GI; 17% vs. 11%	Tumor bed, regional node (nos. 1–16)	87%
2012 IMRT China [27]	45	CRT, 45Gy, 5FU+LV	Similar toxicity mostly well tolerated	Tumor bed, regional node (nos. 1–16)	91%

*CRT* chemoradiotherapy, *CT* chemotherapy, *5-FU* 5-fluorouracil, *LV* leucovorin, *XP* capecitabine plus cisplatin, *2D* 2-dimentional irradiation, *3D* 3-dimensional conformal radiation therapy, *IMRT* intensity-modulated radiation therapy

Yoon retrospectively analysed the follow-up records from 91 stage III gastric carcinoma patients with the N3 disease, who were diagnosed with the first regional relapse after D2 dissection [46]. This study suggested that vessel-based delineations of rnGTVs (recurrent nodal gross tumor volume) on CT images depend on the recurrent sites of LNs from the follow-up records after D2 lymphadenectomy. The results showed that no. 16a (58.2%) and 16b (61.5%) were the most commonly affected first recurrent LNs. In addition, node nos. 9, 12, 13, and 14 were involved in 15.4%, 28.6%, 15.4%, and 19.8% of patients, respectively. Conversely, node nos. 11 (7.0%), 8 (3.0%), 2 (2.0%), and 10 (1.0%) were less commonly involved. When tumor involved the proximal third of stomach, the most commonly involved LNs were nos. 9 (30%), 10 (10%), and 13 (10%) lymph nodes. Nos. 12 and 14 were the most commonly involved LNs, when tumor involved the middle third stomach (26% and 13%, respectively). When tumor involved the distal third stomach, nos. 12, 13, 14, 9, and 11 were the commonly involved metastatic LNs (39%, 27.0%, 20.0%, 20.0%, and 10%, respectively). Nos. 14, 12, 11, 9, and 2 were the commonly involved metastatic LNs (41%, 24.0%, 12%, 12%, and 12%, respectively), when tumor involved more than two-thirds of the stomach. It showed that, in this study, the recurrent sites of lymph nodes such as splenic hilum, perigastric area, and below IMA were uncommon. The treatment volume can exclude the liver hilum (no. 12), perigastric area (nos. 1–6), and anterior part of the SMA (no. 14) when tumor involved the proximal third of the stomach; nevertheless, if CTV encompassed the splenic hilum (no. 10), it should also contain the splenic artery region. The treatment volume should include the perigastric region (nos. 1–6), the splenic hilum (no. 10), and the splenic artery region (no. 11) in the middle or distal third stomach. In addition, when patients with extensive tumor involved more than two-thirds of the stomach were only treated with subtotal gastrectomy, no. 2 LNs should be contained in CTV.

Current studies recommended that the target volume for postoperative RT in gastric cancer covered all nodal recurrence sites. Node nos. 1–6, 10, and 11 can be excluded from the treatment volume because of the extremely low recurrence rate after surgery. Jeong reassessed the ARTIST trial depending on the patterns of postoperative recurrence and the definition of the target volume [30]. The study found that the ARTIST trial was similar to the study of Yoon in failure patterns, and the postoperative concomitant radiochemotherapy significantly decreased the recurrence rate of node nos. 16a/b, 13, and 14 compared with chemotherapy alone. This result indicates that RT has advantages in the control of high-risk lymph node.

According to Yoon, if the tumor involved the proximal third stomach, then the lymph nodes for target volume should include 9, 10, 13, and 16a/b. If the gastric cancer involved the middle third stomach, then the extent should include 12, 14, and 16a/b. If the tumor involved the distal third stomach, then the extent should include 9, 11–14, and 16a/b. If the gastric cancer involved more than two-thirds of stomach, then 2, 9, 11, 12, 14, and 16a/b should be included (Table 4). In the present study, a preliminary plan can be recommended. The anastomotic site should be included because of a high rate of recrudesce. Whether the residual stomach should be irradiated remains controversial, and IMRT can reduce the adverse reactions. The tumor bed should be included for the T4 stage. No deal exists either on the extent of lymph nodes for RT.

**Table 4:** Radiation range of lymph nodes after D2 dissection from Yoon

Primary site	Radiation range
Proximal third stomach	9, 10, 13, and 16a/b
Middle third stomach	12, 14, and 16a/b
Distal third stomach	9, 11–14, and 16a/b
More than two-thirds of the stomach	2, 9, 11, 12, 14, and 16a/b

Additionally, a phase II trial from China provides a new method to contouring the target volumes of lymph node for postoperative RT in gastric cancer [47]. Compared with the traditional surgical-based division system, the stomach is segmented into the upper third-fundus, the middle third-body, and the lower third-pylorus. They advised that the target volumes should always contain the perigastric LNs (nos. 1–6) and the lymphatics in the gastric wall and the LNs around the celiac artery. The other LNs should be irradiated on the basis of their lymphatic drainage.

A preliminary plan from our hospital advises the following points. The gastric cancer involving the proximal third stomach should include 110, 20, 1–3, 7–11, and 16a/b. The gastric cancer involving the middle third stomach should include 1, 3, 5, 9, 11p, 12, 13, 14 (T4 or pancreas involved), and 16a/b. The gastric cancer involving the distal third stomach should include 3, 5, 9, 11p, 12, 13, 14 (T4 or pancreas involved), and 16a/b (Table 5). The effect of this plan has not been reported, and researchers can enlighten the updating path of delineating lymph node target volumes.

**Table 5:** Radiation range of lymph nodes after D2 dissection from the Chinese Academy of Medical Sciences

Primary site	Radiation range
Proximal third stomach	110, 20, 1–3, 7–11, and 16a/b
Middle third stomach	1, 3, 5, 9, 11p, 12, 13, 14*, and 16a/b
Distal third stomach	3, 5, 9, 11p, 12, 13, 14*, and 16a/b

*T4 or pancreas involved

An improved plan for target volumes delineation should be on the basis of clinical experience and the characteristics of lymphatic drainage. Currently, the study of Yoon et al. is the only research on delineating the rnGTV, but they analyzed only the patients with stage III (N3) gastric cancer. The study of Yu provides a new idea for delineating lymph node target volumes; however, the research is single-arm, phase II, and non-randomized. Phase III trials are still necessary to validate the conclusion. The guidelines for the delineation of target volumes for postoperative RT entail further consensus.

## Conclusion

Preoperative RT has progressed in treating GEJ cancer; however, the application of preoperative RT still lacks large-scale phase III clinical trials for gastric cancer. In addition, patients with D1 or D1 plus lymphadenectomy can benefit from postoperative RT obviously, and postoperative RT may be beneficial for some patients with D2 lymphadenectomy. Multicenter randomized controlled trials are still required to confirm the value of RT in patients with this disease.

RT is a promising prospect as a local treatment option; future efforts should be directed to defining the target volume, determining the optimal multimodality protocol, and improving the technology of RT. Screening for novel biomarkers of radiosensitivity will also help patients of gastric cancer benefit from personalized therapy.

## Abbreviations

2D: 2-Dimentional irradiation
3D: 3-Dimensional conformal radiation therapy
5-FU: 5-Fluorouracil
CRT: Chemoradiotherapy
CT: Chemotherapy
DFS/RFS: Disease-/relapse-free survival
EC: Esophagus cancer
EGJ: Esophagogastric junction
GC: Gastric cancer
GEJ: Gastroesophageal junction
IMRT: Intensity-modulated radiation therapy
LNs: Lymph nodes
LRRFS: Locoregional failure-free survival
LV: Leucovorin
NR: Not reported
OS: Overall survival
RT: Radiotherapy
S: Surgery
XP: Capecitabine plus cisplatin
